# Mitochondrial lipid peroxidation is necessary but not sufficient for induction of ferroptosis

**DOI:** 10.3389/fcell.2024.1452824

**Published:** 2024-09-11

**Authors:** He Huan, Konstantin G. Lyamzaev, Alisa A. Panteleeva, Boris V. Chernyak

**Affiliations:** ^1^ Belozersky Institute of Physico-Chemical Biology, Lomonosov Moscow State University, Moscow, Russia; ^2^ The “Russian Clinical Research Center for Gerontology” of the Ministry of Healthcare of the Russian Federation, Pirogov Russian National Research Medical University, Moscow, Russia

**Keywords:** ferroptosis, ferric ammonium citrate (FAC), buthionine sulfoximine (BSO), mitochondrial lipid peroxidation, mitochondrial-targeted antioxidants

## Abstract

Ferroptosis, a form of regulated cell death mediated by lipid peroxidation (LPO), has become the subject of intense research due to its potential therapeutic applications in cancer chemotherapy as well as its pathophysiological role in ischemic organ injury. The role of mitochondrial lipid peroxidation (LPO) in ferroptosis remains poorly understood. We show that supplementation of exogenous iron in the form of ferric ammonium citrate (FAC) in combination with buthionine sulfoximine (BSO, an inhibitor of glutathione biosynthesis) induces mitochondrial lipid peroxidation that precedes ferroptosis in normal human fibroblasts. The mitochondrial-targeted antioxidant SkQ1 and the redox mediator methylene blue, which inhibits the production of reactive oxygen species (ROS) in complex I of the mitochondrial electron transport chain, prevent both mitochondrial lipid peroxidation and ferroptosis, but do not affect the cytosolic ROS accumulation. These data indicate that mitochondrial lipid peroxidation *is required* for ferroptosis induced by exogenous iron. FAC in the absence of BSO stimulates mitochondrial peroxidation without reducing cell viability. Glutathione depletion by BSO does not affect FAC-induced mitochondrial LPO but strongly stimulates the accumulation of ROS in the cytosol. These data allow us to conclude that mitochondrial LPO *is not sufficient* for ferroptosis and that cytosolic ROS mediates additional oxidative events that stimulate ferroptosis in conjunction with mitochondrial LPO.

## Graphical Abstract

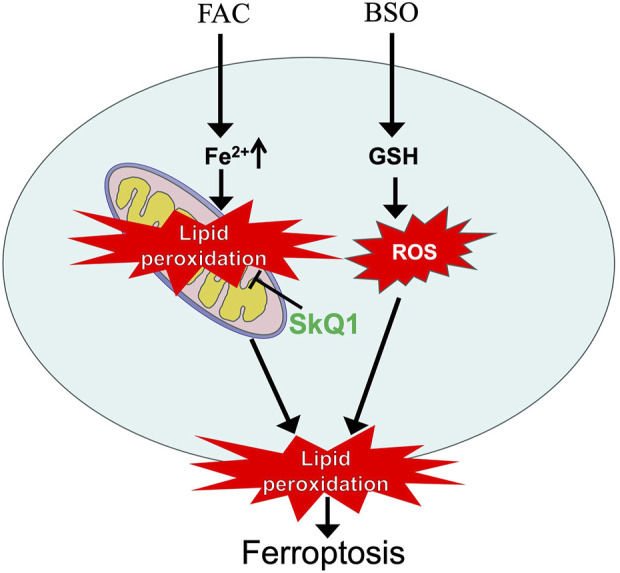

## 1 Introduction

The involvement of mitochondria in ferroptosis has been the subject of debate since ferroptosis has been recognized as a specific form of regulated cell death. Pioneering work by Dixon et al. (2012) reported that mitochondrial DNA depletion had no significant effect on sensitivity to ferroptosis induced by the cystine transport inhibitor erastin. A more recent study (Gao et al., 2019) showed that cells completely depleted of mitochondria by activation of mitochondria-targeted autophagy were less sensitive to ferroptosis induced by cysteine starvation, but not by inhibition of glutathione peroxidase 4 (GPx4), a lipid peroxide detoxifier. It was concluded that mitochondrial metabolism may regulate ferroptosis by modulating the level of reduced glutathione. More recently, it was shown (Gao et al., 2019) that depletion of mitochondrial DNA using the same procedure as in (Dixon et al., 2012) results in increased expression of mitochondrial GPx4 and resistance to erastin-induced ferroptosis. Studies using various mitochondria-targeted antioxidants have provided compelling evidence for the role of mitochondrial ROS in ferroptosis. Conjugates of the triphenylphosphonium cation with ubiquinol (MitoQ) or piperidine nitroxide TEMPO (MitoTEMPO) (Oh et al., 2022), as well as XJB-5-131 (TEMPO conjugated with gemigramicidin S) (Krainz et al., 2016), have been shown to inhibit ferroptosis induced by erastin or the Gpx4 inhibitor RSL3, whereas their untargeted counterparts were much less effective.

In our previous study, we analyzed lipid peroxidation (LPO) in mitochondria during ferroptosis using a novel fluorescent ratiometric probe targeting mitochondria, MitoCLox (Lyamzaev et al., 2023).The specific oxidation of MitoCLox by lipid radicals, its selective accumulation in the mitochondria of various cells and the response to mitochondrial lipid peroxidation were shown in our earlier works (Lyamzaev et al., 2019; Chernyavskij et al., 2023; Lyamzaev et al., 2024). Mitochondrial lipid peroxidation has been shown to precede cell death in models of ferroptosis induced by erastin in SV40-transformed fibroblasts and by the gamma-glutamyl cysteine synthetase inhibitor buthionine sulfoximine (BSO) in fibroblasts from the patients with Leber hereditary optic neuropathy (LHON). Mitochondrial-targeted antioxidants SkQ1 [10-(6′-plastoquinonyl) decyltriphenylphosphonium bromide] and MitoTEMPO inhibit both mitochondrial LPO and ferroptosis. The redox cycling agent methylene blue (MB), which targets mitochondria due to its positive charge, bypasses electron flow past complex I of the electron transport chain and inhibits mitochondrial ROS production, also suppresses mitochondrial LPO, and protects against ferroptosis in two models. Neither SkQ1 nor MB affects cytosolic ROS accumulation as measured by CM-H2DCFDA. These data allow us to conclude that mitochondrial LPO *is necessary* for ferroptosis (Lyamzaev et al., 2023).

In the present study, we examined oxidative stress induced by exogenous iron in normal human fibroblasts to answer the question of whether mitochondrial LPO is *sufficient* to induce ferroptosis.

## 2 Materials and methods

### 2.1 Chemicals

SkQ1 and dodecyltriphenylphosphonium bromide (C_12_TPP) were kindly provided by the Institute of Mitoengineering, Lomonosov Moscow State University. C11-BODIPY581/591 was from Lumiprobe (Moscow, Russia), CM-H2DCFDA was from Invitrogen Life Technologies (Waltham, MA, United States). MitoCLox was synthesized from succinimidyl ester of C11-BODIPY581/591 and (5-[(4-aminobutyl) amino]-5-oxopentyl) triphenylphosphonium bromide as described in (Lyamzaev et al., 2019). MitoCLox, a ratiometric fluorescent dye that specifically reacts with lipid peroxy radicals, is addressed to mitochondria by conjugation of the fluorophore C11-BODIPY581/591 with the penetrating triphenylphosphonium cation. MitoCLox was shown to selectively accumulates in the mitochondria of various living cells and registers mitochondrial lipid peroxidation (Lyamzaev et al., 2020; Chernyavskij et al., 2023). Other reagents, except for those indicated, were from Sigma-Aldrich (Saint Louis, MO, United States).

### 2.2 Cell cultures

Human primary skin fibroblasts from Common Use Center “Biobank” (Research Centre for Medical Genetics, Moscow, Russia) were cultured in DMEM (Dulbecco’s modified Eagle’s) medium (Gibco; Thermo Fisher Scientific, Inc., Waltham, MA, United States) supplemented with 2 mM glutamine and 10% fetal bovine serum (FBS) (HyClone, Logan, UT 84321 United States) and 100 U/mL streptomycin and 100 U/mL penicillin (all from Gibco, United States). Cells were challenged with 0.6 mM FAC alone or in combination with 1 mM BSO for 24 h or 48 h. Where indicated 0.1 mM ferrostatin-1, 0.2 mM Trolox, 10, 50 nM SkQ1, 50 nM C12TPP, or 250 nM MB were added concomitantly with FAC. Cell viability was measured using the CellTiterBlue^®^ reagent (Promega, United States) according to the manufacturer’s protocol with Fluoroskan Ascent FL Microplate Reader (Thermo Labsystems, Waltham, MA, United States).

### 2.3 Microscopy

Fibroblasts were plated in 35 mm glass bottom (SPL) dishes for confocal microscopy at 150,000 cells. After incubation with ferric ammonium citrate (FAC) for 24 h, cells were stained with 50 μg/mL propidium iodide and 8 μM Hoechst 33,258 for 30 min. Image acquisition was performed using a fluorescence microscope Olympus IX 83 (Japan).

### 2.4 Flow cytometry

Fibroblasts were stained with 100 nM MitoCLox (1 h), or 2 μM C11-BODIPY581/591 (30 min), or 1.8 µM CM-H2DCFDA (30 min). Cells were stripped with trypsin/versene, centrifuged in 1.5 mL tubes (900 g, 5 min) at 4°C and redispersed in 30 mL PBS. Flow cytometry analyses were performed using an Amnis FlowSight Imaging Flow Cytometer (Luminex Corporation, Seattle, WA, United States) with excitation at 488 nm and the detection channels 480–560 nm (Ch2) and 595–642 nm (Ch4). Channel 2 (Ch2) was used to detect autofluorescence, which indicates lipofuscin content (Malavolta et al., 2022). Each sample was measured until 4,000 events were collected. For ratiometric analysis, the Amnis IDEAS^®^ 6.2 (Luminex, Seattle, WA, United States) image analysis software was used. Data are presented as geometric means computed using flow cytometry software.

### 2.5 Measurements of labile iron pool

Intracellular labile iron was measured using acetoxymethyl ester of calcein (calcein-AM) as described elsewhere (Breuer et al., 1995). This dye is initially non-fluorescent, becoming fluorescent after enzymatic modification upon penetrating the cell membrane. The fluorophore then stoichiometrically binds to iron, which quenches its green fluorescence (Tenopoulou et al., 2007). For the experiment, cells were seeded onto a 96-well plate (10 × 10³ cells per vial) and incubated with varying concentrations of FAC for 48 h with or without SkQ1 (50 nM). To chelate free iron, cells were incubated with 1 mM deferroxamin (DFO) for 2 h before the addition of calcein. After this the cells were washed with serum-free medium, followed by the addition of 200 nM calcein-AM for 20 min. Post-incubation, the cells were washed three times with Hanks medium, and the fluorescence of calcein was measured using a Fluoroskan Ascent FL Microplate Reader (Thermo Labsystems, Waltham, MA, United States) with excitation/emission settings of Ex485/Em538. To normalize the cell count in each well, the CellTiterBlue^®^ reagent (Promega, United States) was used, following the manufacturer’s protocol.

### 2.6 Statistics

At least three repeats for each measurement were performed. Results are presented as the mean of a minimum of 3 independent replicates with standard deviation (SD). Comparisons were analyzed by one-way ANOVA. The significance was analyzed with Prism 10.0 software (GraphPad Software, LLC, California, United States); a value of *p* < 0.05(** or #) was considered to be statistically significant.

## 3 Results

**FIGURE 1:**
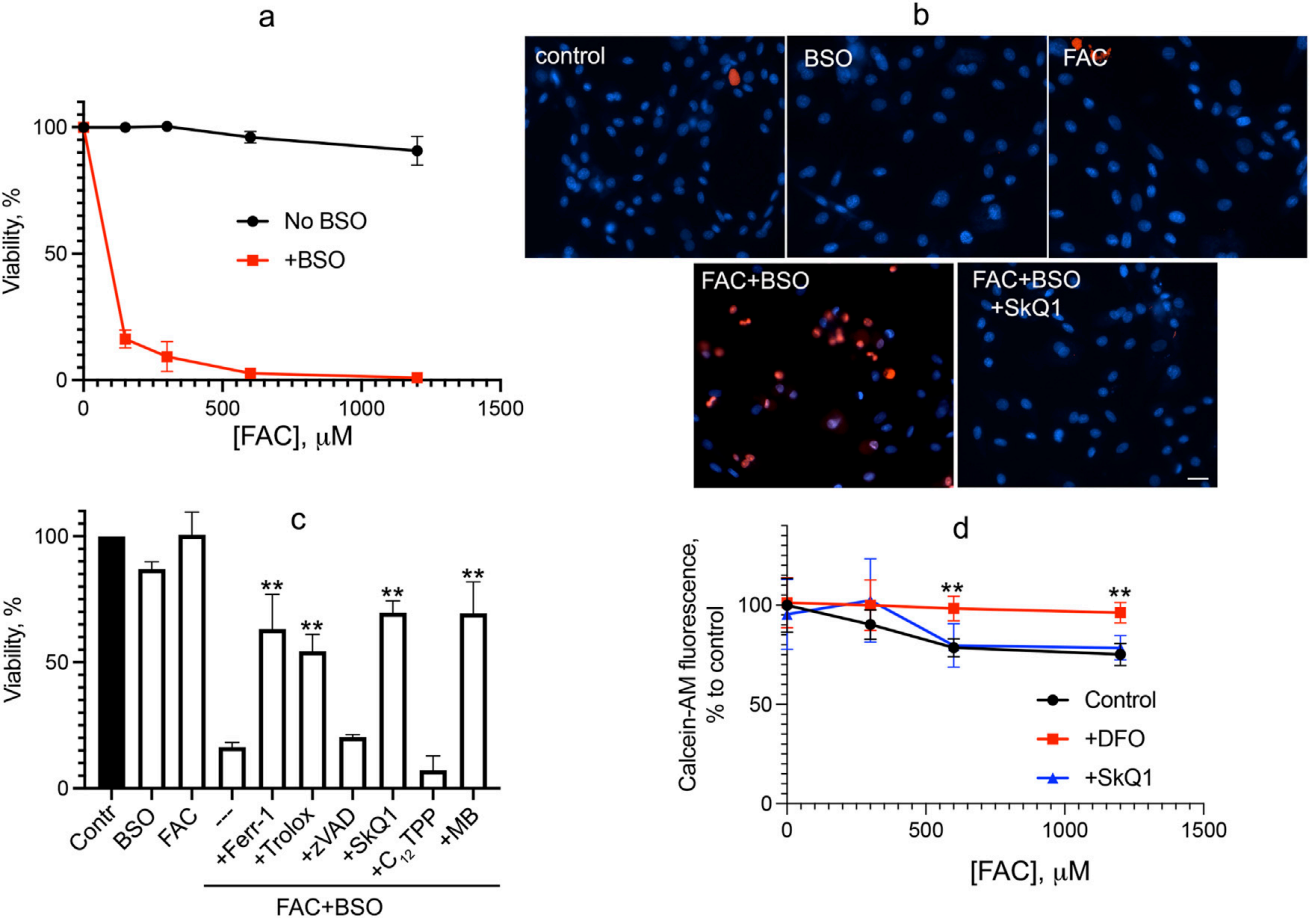
Glutathione depletion by BSO promotes ferroptosis induced by FAC in human fibroblasts. **(A)** Cells were incubated with FAC alone or in combination with 1 mM BSO for 48 h. Cell viability was measured using the CellTiterBlue reagent. **(B)** Cells were incubated with 0.6 mM FAC and 1 mM BSO separately or in combination for 48 h and stained with 50 μg/mL propidium iodide (red) and 8 μM Hoechst 33,258 (blue) for 30 min 50 nM SkQ1 was added where indicated together with FAC + BSO. Cells were analyzed using Olympus IX83 fluorescent microscope. Bar 20 μm. **(C)** Viability of cells incubated with 0.6 mM FAC alone or in combination with 1 mM BSO for 48 h. Where indicated 0.1 mM ferrostatin-1 (Ferr-1), 0.2 mM Trolox, 10 μM zVADfmk (zVAD), 50 nM SkQ1, 50 nM C_12_TPP, 250 nM MB were added. *p* < 0.05(**)—the significance of the difference between samples treated with FAC + BSO and other samples. **(D)** Labile iron pool was measured as the quenching of intracellular calcein fluorescence after 48 h of incubation with FAC or FAC with 50 nM SkQ1. Where indicated, 1 mM deferoxamine (DFO) was added 2 h prior to сalceine-AM loading. *p* < 0.05(**) - the significance of the difference between samples with DFO and other samples.

We analyzed oxidative stress caused by iron overload supplying ferric ammonium citrate (FAC), which is a physiological form of non-transferrin-bound iron widely used as a dietary supplement. FAC has low toxicity but has been shown to sensitize HT-1080 fibrosarcoma cells to erastin-induced ferroptosis (Dixon et al., 2012). As shown in Figure 1, FAC does not induce cell death in normal human fibroblasts at concentrations up to 1.2 mM. Glutathione depletion by BSO is not toxic but strongly promotes fibroblast cell death induced by FAC (Figure 1A). Cell death induced by combination of FAC and BSO is necrotic, as detected by propidium iodide staining of nuclei (Figure 1B), and is not prevented by the pan-caspase inhibitor zVADfmk (Figure 1C), so secondary caspase-dependent necrosis is excluded. The ferroptosis inhibitor ferrostatin-1 (fer-1) and another antioxidant, the water-soluble vitamin E analogue Trolox, prevent a decrease in viability. Mitochondrial-targeted antioxidant SkQ1 protects against ferroptosis induced by the combination of FAC and BSO at very low concentrations, whereas SkQ1 analogue lacking the antioxidant moiety dodecyltriphenylphosphonium (C_12_TPP) is ineffective (Figure 1C). Importantly, SkQ1 was shown to have no effect on the increase in intracellular labile iron pool (LIP) induced by 48 h incubation with FAC (Figure 1D). These data indicate that ROS-dependent mitochondrial processes are critical for ferroptosis induced by exogenous iron. Methylene blue (MB), which inhibits mitochondrial complex I–dependent ROS production, also protects against ferroptosis induced by combination of FAC and BSO (Figure 1C) indicating that FAC-induced mitochondrial ROS production is at least partially originated from complex I.

Cell death induced by FAC combined with BSO was measured after 48 h of incubation, whereas minor changes in cell viability were observed after 24 h of incubation with up to 1.2 mM FAC either alone or in combination with 1 mM BSO. This is why a 24-h incubation was chosen to measure the oxidative event preceding cell death.

**FIGURE 2:**
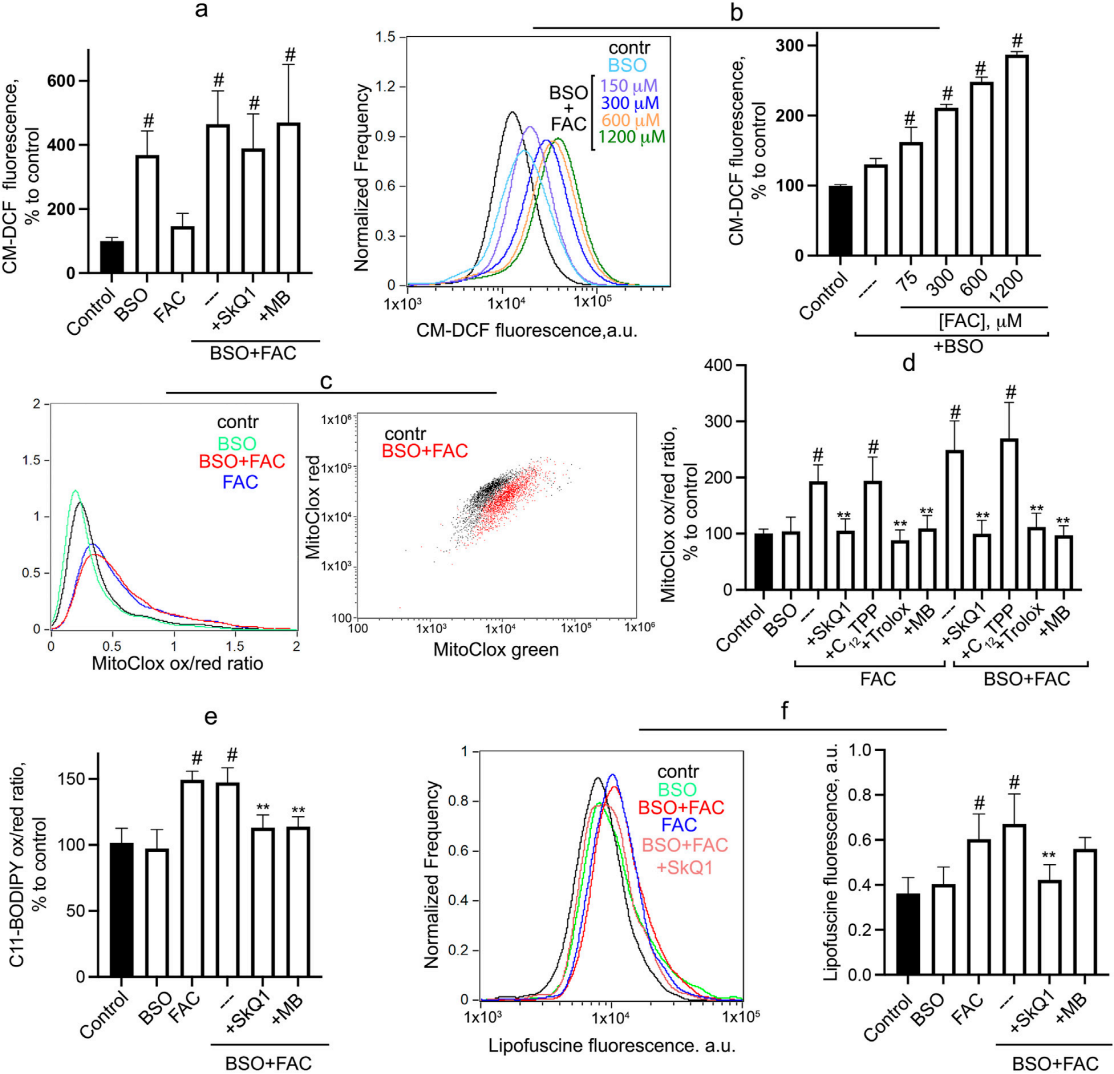
Oxidative events induced by FAC and BSO in human fibroblasts. Cells were incubated with 0.6 mM FAC, BSO (1 mM or 0.3 mM) or with their combination for 24 h **(A)** Cytosolic ROS accumulation was measured using CM-H2DCFDA. Mean values of fluorescence are presented. Cells were challenged with 1 mM BSO alone and in combination with FAC or different concentrations of FAC in combination with 0.3 mM BSO **(B)**. 50 nM SkQ1, 250 nM MB were added at the same time as BSO and FAC where indicated. **(C, D)** Mitochondrial lipid peroxidation was measured using MitoCLox. Cells were incubated with 0.6 mM FAC alone or in combination with 1 mM BSO for 24 h and stained with 100 nM MitoCLox for 1 h. Ratio of green/red fluorescence was measured. Typical histograms **(С)** and mean values **(D)** are shown. 50 nM SkQ1, 50 nM C12TPP, 250 nM MB and 0.2 mM Trolox were added where indicated. **(E)** Total lipid peroxidation was measured using C11-BODIPY581/591. Cells were incubated with 1 mM BSO and 0.6 mM FAC for 24 h and stained with 2 μM C11-BODIPY581/591 for 30 min and analyzed using flow cytometry. Ratio of green/red fluorescence was measured. Mean values are presented. 50 nM SkQ1, 50 nM C_12_TPP, 250 nM MB and 0.2 mM Trolox were added where indicated. **(F)** FAC-induced accumulation of lipofuscin-like material was analyzed using flow cytometry without staining. Cells were incubated with 0.6 mM FAC alone and in combination 1 mM BSO for 24 h. 50 nM SkQ1, 250 nM MB were added at the same time as BSO and FAC. Mean values of fluorescence are presented. *p* < 0.05(**)—the significance of the difference between samples treated with FAC or FAC + BSO and other samples. *p* < 0.05(#)—the significance of the difference between control and other samples.

Cytosolic ROS accumulation, measured by CM-H2DCFDA, is not stimulated by FAC but is significantly increased after 24-h incubation with BSO (Figure 2A). At 1 mM BSO, FAC does not affect the level of cytosolic ROS, whereas at a suboptimal concentration of BSO (0.3 mM), FAC dose-dependently stimulates the accumulation of cytosolic ROS (Figure 2B). SkQ1 and MB do not affect BSO + FAC induced H_2_O_2_ accumulation (Figure 2A), indicating that mitochondrial ROS do not contribute significantly to the induction of general oxidative stress in this model. Similar results were obtained previously for erastin-induced accumulation of cytosolic ROS in fibroblasts (Lyamzaev et al., 2023).

As shown in Figures 2C, D, FAC significantly stimulates mitochondrial LPO, and the addition of BSO does not affect FAC-induced LPO (or does not induce mitochondrial LPO itself). Trolox, SkQ1 (but not C_12_TPP) and MB prevent mitochondrial lipid peroxidation induced by either FAC or FAC + BSO. Since BSO treatment appears to be critical for FAC-dependent cell death (Figure 1), it can be assumed that mitochondrial LPO is not sufficient for ferroptosis. Interestingly, similar effects are observed when measuring total lipid peroxidation using C11-BODIPY581/591 (Figure 2E). BSO does not induce total LPO and does not affect FAC-induced peroxidation. SkQ1 and MB inhibit total LPO induced by combination of FAC and BSO, indicating that (in contrast to H_2_O_2_ accumulation) mitochondrial ROS contribute significantly to total lipid peroxidation.

Lipofuscin, a heterogeneous complex mixture composed of highly oxidized lipids and cross-linked proteins, can be consider as a marker of severe oxidative stress (Terman and Brunk, 2004). Incubation of fibroblasts with FAC for 24 h results in accumulation of lipofuscin-like material, as evidenced by increased autofluorescence in a wide spectral range 480–560 nm (Figure 2F). As in the cases of mitochondrial and total LPO (Figures 2D, E), BSO does not induce lipofuscin accumulation and does not affect FAC-induced accumulation. SkQ1 inhibits the accumulation of lipofuscin-like material induced by combination of FAC and BSO. It is considered a marker of organismal aging and cellular senescence and usually accumulates very slowly (von Zglinicki et al., 1995). The rapid accumulation of lipofuscin-like material in FAC-treated fibroblasts reflects lipid peroxidation and is dependent on mitochondrial ROS production.

## 4 Discussion

As shown in Figure 1, the mitochondria-targeted antioxidant SkQ1 and the redox agent methylene blue, which inhibits ROS production by complex I of the mitochondrial electron transport chain, prevent ferroptosis induced by ferric ammonium citrate in combination with the glutathione biosynthesis inhibitor BSO. Data in Figure 2 show that mitochondrial lipid peroxidation precedes ferroptotic cell death and is prevented by SkQ1 and MB, whereas these mitochondria-targeting agents do not affect cytosolic ROS accumulation. These data indicate that mitochondrial LPO *is required* for ferroptosis induced by exogenous iron. This finding is entirely consistent with results obtained previously for models of ferroptosis induced by erastin in SV40-transformed fibroblasts and BSO in fibroblasts from LHON patients (Lyamzaev et al., 2023).

Evidence for the contribution of mitochondrial iron-dependent oxidative events to ferroptosis was provided also by studies in which overexpression of the mitochondrial ferritin isoform (MtFt) was shown to inhibit ferroptosis induced by erastin (Wang et al., 2016), as well as by doxorubicin or simulated ischemia/reperfusion (Chen et al., 2023). Ferritin is known to sequester labile iron, inhibiting the formation of hydroxyl radicals in the Fenton reaction and lipid peroxidation, so these data indicate an important role for mitochondrial lipid peroxidation in ferroptosis. Recently, important studies have identified the role of the natural mitochondrial lipophilic antioxidant coenzyme Q (CoQ) in protection against ferroptosis. Two enzymes localized to the inner mitochondrial membrane, dihydroorotate dehydrogenase (DHODH) (Mao et al., 2021) and glycerol-3-phosphate dehydrogenase 2 (GPD2) (Wu et al., 2022), have been reported to inhibit mitochondrial lipid peroxidation and protect against ferroptosis by reducing of CoQ to CoQH2, which can detoxify lipid peroxyl radicals in mitochondria. However, it should be noted that studies of the protective effects of DHODH have recently come under severe criticism based on experimental data (Mishima et al., 2023).

In contrast to the effects of erastin, the use of FAС allows the separation of mitochondrial lipid peroxidation and ferroptosis in fibroblasts. As shown in Figure 2, FAC stimulates mitochondrial LPO without reducing cell viability. Glutathione depletion by BSO promotes FAC-induced ferroptosis, presumably by reducing GPx4 activity and stimulating plasma membrane LPO. At the same time, BSO does not have a significant effect on FAC-induced mitochondrial LPO. These data allow us to conclude that mitochondrial LPO is *not sufficient* for ferroptosis. In contrast to FAC, BSO strongly stimulates cytosolic ROS accumulation (Figure 2A), suggesting that cytosolic ROS mediates additional oxidative events that stimulate ferroptosis in conjunction with mitochondrial LPO. SkQ1 and MB do not affect BSO-induced cytosolic H_2_O_2_ accumulation, so the second ferroptotic stimulus in this model is independent of mitochondrial ROS.

Interestingly, FAC induces significant total lipid peroxidation that does not lead to fibroblasts death. BSO neither induces total LPO nor stimulates FAC-induced peroxidation. Thus, general LPO, which develops in all cell membranes, cannot always be considered as a marker of ferroptosis. Different cell types have different sensitivities to FAC-induced cell death. For example, in a recent study ferroptosis was observed in islet β-cells treated with submillimolar concentrations of FAC (Deng et al., 2023). The possible role of mitochondrial LPO in ferroptosis of islet β-cells, as well as the nature of the difference in sensitivity to exogenous iron between these cells and fibroblasts, deserves further study. We also described FAC-induced ferroptosis associated with mitochondrial LPO in cardiomyocytes (Lyamzaev et al., 2024). The conclusion reached in our study concerns the basic mechanisms of ferroptosis, and it would be important to demonstrate its validity in other cell models with other ferroptosis inducers. The role of iron overload in induction of ferroptosis involved in various pathologies is well known (Chen et al., 2023; Ryan et al., 2023), while the mechanisms of ferroptosis caused by exogenous iron are still poorly understood. Since SkQ1 and MB effectively inhibit FAC-induced ferroptosis, mitochondria-targeted agents may be considered candidates for the treatment of various pathologies associated with iron overload. At the same time, our data show that mitochondrial oxidative events are not sufficient to induce ferroptosis and additional cytosolic ROS-dependent mechanisms should be taken into account.

## Data Availability

The original contributions presented in the study are included in the article/supplementary material, further inquiries can be directed to the corresponding authors.
